# Case of Chloroformization in Midwifery

**Published:** 1855-09

**Authors:** 


*Case of Chloroformization in Midwifery.*

Dr. M'Clintock briefly related the history of a case of Chloroformization in Midwifery Practice, in order to show the great necessity that existed for always using the utmost caution and circumspection in the employment of cholorofrom inhalation in labor, as well as the great importance of intrusting its exhibition to none but a medical man. He did so because the writings and practice of some of the foremost advocates for anæsthesia had led many persons to suppose that the administration of chloroform to parturient cases was almost, if not entirely, free from danger,—a most dangerous fallacy, and one that would inevitably lead to disastrous consequences if generally acted on.

The leading circumstances of the case he brought forward were these: —A healthy woman, aged 28, was admitted into the Lying-in Hospital in labor of her first child, some days before. The first stage was very tedious in consequence of an unyielding condition of the os uteri, and much general irritability. With a view to relieve this state, and to give her some rest, as she was much harassed by frequent short pains, it was thought advisable to put her under the influence of chloroform. For about an hour she got it in small quantities,—in fact, merely *chloroform à la reine*,—but without experiencing any benefit, and without its producing any anæsthetic or soporific effect. The quantity put on the sponge, a large cup-shaped one, was now increased, but still could hardly have exceeded one drachm, and was not more than is habitually given in the hospital to patients undergoing obstetric operations, to which cases its use is chiefly limited; and it was administered by the senior assistant, who has had ample experience of its use, having given it before in hundreds of instances. After the sponge was reapplied to her mouth, and she had taken three or four inspirations, a change came over her countenance, the eyeballs turned up, the pulse left the wrist, respiration was suspended for a space of time that would have occupied about three or four inspirations, and some froth collected at the angles of the mouth. On the first appearance of these alarming symptoms the sponge was instantly withdrawn, the free circulation of fresh air was promoted, the face and chest were aspersed with cold water, and ammonia was applied to the nostrils. It should, perhaps, be mentioned, that during the entire of the above period she was in bed, and lying down. Under the influence of these restoratives animation gradually returned. It was evident, however, to all around—and many of the pupils were present—that she was all but gone, and that her life was preserved by the early recognition of the poisonous effects of the medicine, and the prompt and judicious employment of appropriate restoratives. Hardly any one will venture to deny that had this woman been in non-professional hands, her life would have been lost. That she got a dose of chloroform, which

*to her* was an overdose, is sufficiently obvious; and yet the quantity given, the quality of the chloroform used, the mode of exhibition, and even the administrator, were all the same as on hundreds of previous occasions, when everything went on favorably. Hence, then, the absolute necessity for *invariably and uniformly observing the strictest caution, prudence, and circumspection in the employment of this powerful agent, and never intrusting its exhibition to a non-medical person.* A case that strikingly illustrates these remarks is recorded in the Medical Times and Gazette for April 14, 1855, where a lady died in the course of a natural labor from the effects of chloroform administered to her by the nurse, on a handkerchief, without the sanction or knowledge of the doctor, who was in the house at the time. The quantity used in this case, with fatal effect, could not have exceeded five fluid drachms.—*Dublin Quarterly Journal—Transactions of the College of Physicians in Ireland.*

